# Integrating multiple machine learning methods to construct glutamine metabolism-related signatures in lung adenocarcinoma

**DOI:** 10.3389/fendo.2023.1196372

**Published:** 2023-05-17

**Authors:** Pengpeng Zhang, Shengbin Pei, Leilei Wu, Zhijia Xia, Qi Wang, Xufeng Huang, Zhangzuo Li, Jiaheng Xie, Mingjun Du, Haoran Lin

**Affiliations:** ^1^ Department of Thoracic Surgery, The First Affiliated Hospital of Nanjing Medical University, Nanjing, China; ^2^ Department of Breast Surgery, The First Affiliated Hospital of Nanjing Medical University, Nanjing, China; ^3^ Department of Radiation Oncology, Shanghai Pulmonary Hospital, Tongji University School of Medicine, Shanghai, China; ^4^ Department of General, Visceral, and Transplant Surgery, Ludwig-Maximilians-University Munich, Munich, Germany; ^5^ Department of Gastroenterology, Affiliated Hospital of Jiangsu University, Jiangsu University, Zhenjiang, China; ^6^ Faculty of Dentistry, University of Debrecen, Debrecen, Hungary; ^7^ Department of Cell Biology, School of Medicine, Jiangsu University, Zhenjiang, China; ^8^ Department of Burns and Plastic Surgery, The First Affiliated Hospital of Nanjing Medical University, Nanjing, China

**Keywords:** lung adenocarcinoma, glutamine, signature, prognosis, machine learning

## Abstract

**Background:**

Glutamine metabolism (GM) is known to play a critical role in cancer development, including in lung adenocarcinoma (LUAD), although the exact contribution of GM to LUAD remains incompletely understood. In this study, we aimed to discover new targets for the treatment of LUAD patients by using machine learning algorithms to establish prognostic models based on GM-related genes (GMRGs).

**Methods:**

We used the AUCell and WGCNA algorithms, along with single-cell and bulk RNA-seq data, to identify the most prominent GMRGs associated with LUAD. Multiple machine learning algorithms were employed to develop risk models with optimal predictive performance. We validated our models using multiple external datasets and investigated disparities in the tumor microenvironment (TME), mutation landscape, enriched pathways, and response to immunotherapy across various risk groups. Additionally, we conducted *in vitro* and *in vivo* experiments to confirm the role of LGALS3 in LUAD.

**Results:**

We identified 173 GMRGs strongly associated with GM activity and selected the Random Survival Forest (RSF) and Supervised Principal Components (SuperPC) methods to develop a prognostic model. Our model’s performance was validated using multiple external datasets. Our analysis revealed that the low-risk group had higher immune cell infiltration and increased expression of immune checkpoints, indicating that this group may be more receptive to immunotherapy. Moreover, our experimental results confirmed that LGALS3 promoted the proliferation, invasion, and migration of LUAD cells.

**Conclusion:**

Our study established a prognostic model based on GMRGs that can predict the effectiveness of immunotherapy and provide novel approaches for the treatment of LUAD. Our findings also suggest that LGALS3 may be a potential therapeutic target for LUAD.

## 1 Introduction

Based on the latest global cancer statistics, lung cancer (LC) remains the leading cause of cancer-related mortality worldwide, with more than 350 deaths per day in 2022, despite the acceleration in the decline of its morbidity and mortality (1). Non-small cell lung cancer (NSCLC) and small cell lung cancer (SCLC) are the two major subtypes, with NSCLC accounting for approximately 80% of all LC cases. Among NSCLC, LUAD is the most frequently observed histological subtype (2). Although treatment options including surgery, chemotherapy, radiotherapy, and chemoradiotherapy have continuously advanced, the clinical outcomes for LC patients remain unfavorable, with a 5-year survival rate of only approximately 21% (3). In light of the rapidly developing field of precision medicine, novel strategies, particularly immunotherapies and targeted therapies, have been proposed as potential means of extending the survival of LUAD patients.

Metabolism is a fundamental process that governs cellular functions. The Warburg effect, a well-established phenomenon, has provided ample evidence to support the critical role of metabolism in malignant cell proliferation. Glutamine (Gln), a non-essential amino acid, plays a unique nutritional role in cancer cell proliferation by contributing carbon and nitrogen to a series of growth-promoting pathways (4). While glucose serves as the primary energy source for tumor cell metabolism, glutamine is also essential and is known as “glutamine addiction” (5). Glutamate can directly contribute to the biosynthesis of proline and glutathione, which are important intracellular antioxidant molecules, or it can be deaminated to α-ketoglutarate, which acts as a carbon source to replenish the tricarboxylic acid (TCA) cycle. However, this results in the formation of a hypoxic and acidic tumor microenvironment (TME), which is unfavorable to antitumor immune responses (6, 7). Glutamine addiction plays a crucial role in acquired drug resistance and metastasis of NSCLC and targeting the glutamate dehydrogenase 1 (GLUD1) pathway may provide a promising therapeutic strategy for NSCLC (8). Therefore, we hypothesize that inhibiting glutamine metabolism could limit tumor growth and facilitate the restoration of antitumor immunity. However, the therapeutic efficacy of single-targeted blockade of glutaminase against tumors is generally limited.

In recent years, an increasing number of researchers have turned to combined therapies for the treatment of a variety of cancers, including but not limited to prostate cancer, breast cancer, and ovarian cancer (9–11). While several therapies have shown initial promise, most are still in the clinical trial stage. Recent studies have identified crucial transcription factors that regulate glutamine metabolism (GM) in LUAD cells, such as NRF2. These transcription factors can activate the expression of genes involved in glutamine uptake and metabolism, thereby promoting the development and progression of LUAD. Gaining a deeper understanding of the mechanisms underlying GM in LUAD cells could provide valuable insights into the development of new therapeutic strategies (12).

Artificial intelligence (AI) comprises of a range of technologies that aim to replicate human intelligence using computing systems. Machine learning (ML), a subset of AI, utilizes mathematical algorithms to identify patterns in data and is utilized to make predictions. ML has demonstrated significant efficacy across diverse fields, including wireless communication, speech recognition, and search engines (13, 14). There is mounting evidence suggesting that AI and ML have the potential to aid clinicians in improving clinical diagnosis and treatment decisions or even supplant human judgment (15, 16). With the increasing application of genomics in healthcare, it is anticipated that AI and ML will become more widespread tools in facilitating precision oncology in this digital age.

The objective of this study was to identify GMRGs in LUAD and elucidate their role in the tumor immune microenvironment and prognosis of LUAD. Our findings have the potential to enhance the precision of glutamine-dependent therapeutic schedules, offering new perspectives on prognostic biomarkers and therapeutic targets for LUAD.

## 2 Materials and methods

### 2.1 Data sources

We obtained scRNA-seq data for LUAD from the Gene Expression Omnibus (GEO) database (accession number GSE150938), which comprised 12 LUAD samples. For the training cohort, we obtained LUAD RNA expression patterns and relevant clinical data from The Cancer Genome Atlas (TCGA) database. To validate our findings, we downloaded expression profiles from eight GEO datasets, including GSE13213 (n=117), GSE26939 (n=115), GSE29016(n=39), GSE30219 (n=85), GSE31210 (n=226), GSE37745 (n=106), GSE42127 (n=133), and GSE68465 (n=442). To ensure comparability across datasets, all expression data was normalized to transcripts per million (TPM), and batch effects were removed using the “sva” package (17). Prior to analysis, all data was log2 transformed. We identified GMRGs using the GeneCards database and selected 141 GMRGs with correlation scores greater than 15 for further study.

### 2.2 Flow of scRNA-seq data analysis

The accuracy of the scRNA-seq data was verified using the “Seurat” R package (18, 19), with the following screening criteria: genes expressed in at least three cells, 200-7000 genes expressed in each cell, and less than 10% mitochondrial gene expression. A total of 46,286 suitable cells were identified. The “FindVariableFeatures” tool was used to identify the top 3000 highly variable genes. Batch effects that could interfere with downstream analysis were removed using the “findintegrationanchors” function of the canonical correlation analysis (CCA). To properly integrate and expand the data, the “IntegrateData” and “ScaleData” functions were utilized. Principal component analysis (PCA) was conducted to determine anchor points, followed by t-distribution random neighborhood embedding (t-SNE) algorithm testing on the first 20 principal components to identify significant clusters (20). Using the “FindNeighbors” and “FindClusters” functions (resolution = 0.8), we obtained 20 cell clusters. The cell cycle heterogeneity along the cell clusters was assessed using cell cycle markers included in the “Seurat” package. Cell cycle scores were determined using the “CellCycleScoring” tool, based on the expression of G2/M- and S-phase markers. Differentially expressed genes (DEGs) for each cluster were identified using the “FindAllMarkers” tool. A cut-off threshold with modified P < 0.01 and log2 (foldchange) > 0.25 criteria were used to determine which genes were used as markers for each cluster. Cell types were identified based on typical marker genes for each cluster. GM activity scores were assigned to each cell using the “AUCell” R package to analyze gene set activity status. The cells were segregated into high- and low-GM-AUC groups based on the median AUC score, and the “ggplot2” R package was used for visualization.

### 2.3 Acquiring key genes for regulating GM activity in bulk RNA-Seq

To calculate the absolute enrichment percentage of a particular gene set in each sample, we used ssGSEA (21). In this study, we used ssGSEA to determine the TCGA-LUAD GM enrichment values for each individual. We utilized the “WGCNA” R package as a biological methodology to construct the gene co-expression network (22). The specific procedures were as follows: the tumor samples were pooled, a cut-off line of 120 was established, outliers were removed, and missing value genes were deleted using the “goodSamplesGenes” function. The appropriate soft threshold for adjacency calculation was then visually determined. The expression matrix was transformed into an adjacency matrix and subsequently into a topological overlap matrix (TOM) to determine the genetic connectivity of the network. Average linkage hierarchical clustering was performed based on the variances in TOM. The hierarchical clustering tree was dynamically pruned to identify related modules and to combine modules with strong correlation values (R > 0.25). The module eigengene (ME) was the main component of gene modules, which could substitute for all other genes in a specific module. The correlation between eigengene values and clinical traits was assessed using Pearson correlation. Finally, module genes with the most significant correlation to GM score were selected for further analysis.

### 2.4 Signature produced using integrative machine learning methods

To develop a consensus signature with excellent accuracy and stability performance, we incorporated 10 machine learning algorithms and 117 algorithm combinations. These integrative methods included Lasso, elastic network (Enet), Ridge, stepwise Cox, CoxBoost, partial least squares regression for Cox (plsRcox), RSF, SuperPC, generalized boosted regression modeling (GBM), and survival support vector machine (survival-SVM). The process for creating signatures was as follows: the 173 key genes regulating GM activity were used to fit prediction models based on the leave-one-out cross-validation (LOOCV) framework in the TCGA-LUAD cohort using 117 algorithm combinations (23). All models were evaluated in eight validation datasets (GSE13213, GSE26939, GSE29016, GSE30219, GSE31210, GSE37745, GSE42127, GSE68465).

### 2.5 Model evaluations and nomogram establishment

A heatmap was generated by integrating the model gene expression and clinical features using the R package “pheatmap”. The proportion of clinical stages (Stage I, Stage II, and Stage III-IV) in different risk groups was displayed using a stacked bar chart drawn with the “ggplot2” R package. To better estimate the 1-, 3-, and 5-year OS probability, clinical information (age and clinical stage) and risk scores were integrated to construct a nomogram using the “rms” R package (24). The nomogram’s prediction accuracy was assessed by using the receiver operating characteristic (ROC) curve (25), calibration curve, and concordance index curves.

### 2.6 Assessment of immune infiltration

To further investigate the differences in TME components between different risk groups, we performed gene set enrichment analysis (GSEA) on the hallmark gene sets using the “clusterProfiler” R package (26). Additionally, we compared the mutation landscape of high- and low-risk groups using the “maftools” R package (27) and identified the top 20 mutated genes in each group. Finally, we explored the correlation between risk scores and the response to immunotherapy by analyzing the expression of immune checkpoint genes in different risk groups using the “limma” R package (28).

### 2.7 Mutation landscape

We obtained gene mutation profiles of LUAD patients from the TCGA database and utilized the “ComplexHeatmap” R package to visualize the mutation landscape and immune infiltration scores. Based on the median risk score and the median tumor mutational burden, TCGA-LUAD patients were stratified into four groups, namely H-TMB+high-risk, H-TMB+low-risk, L-TMB+high-risk, and L-TMB+low-risk. We then compared the survival differences among these groups.

### 2.8 Immunotherapy comparisons

Immune checkpoints are a group of molecules expressed on immune cells that regulate the level of immune activation and are critical in limiting excessive immune activation. We compared the expression levels of well-known immune checkpoint genes (ICGs) between the high- and low-risk groups. We also investigated the correlation between ICG expression and both model genes and risk scores. Additionally, we retrieved the Immunophenoscores (IPS) for LUAD from The Cancer Immunome Atlas (TCIA) database (29).

### 2.9 Enrichment analysis

To calculate the enrichment scores of infiltrating immune cells and immunological function, we utilized the ssGSEA method (21, 30). Additionally, to identify enriched GO terms, we analyzed DEGs between the low-risk and high-risk groups using the “clusterProfiler” and “org.Hs.eg.db” R packages (31). GSEA was also employed to determine the signaling pathways and biological activities that were predominantly enriched in both the high- and low-risk groups (32).

### 2.10 Protein interaction network and Core Gene

We utilized the String database to investigate the protein-protein interaction (PPI) network among model genes (33). Furthermore, based on the expression levels of core genes, patients were classified into high- and low-expression groups to compare the differences in survival.

### 2.11 Tissue collection and cell lines culture

Ten paired tissue samples, including tumor tissue (T) and adjacent non-tumor tissue (N), were collected from patients with LUAD who underwent tumor resection after obtaining approval from the Medical Ethics Committee (2019-SR-156). The samples were preserved at -80°C. Human LUAD cell lines A549 and H1299 and normal human lung epithelial cells (BEAS-2B) were obtained from the Cell Resource Center of Shanghai Life Sciences Institute and cultured in F12K or RPMI-1640 (Gibco BRL, USA) supplemented with 10% fetal bovine serum (FBS) and 1% penicillin-streptomycin (Gibco, Invitrogen, Waltham, MA, USA) under 5% CO2, 95% humidity, and 37°C.

### 2.12 Cell transfection

To generate LGALS3 knockdown, small interfering RNAs (siRNAs) were used (34). The siRNA sequences for LGALS3 are listed in **Supplementary Table S1**. In brief, cells were seeded at 50% confluence in a 6-well plate and transfected with negative control (NC) or LGALS3 siRNA using Lipofectamine 3000 (Invitrogen, USA).

### 2.13 Extraction of RNA and real-time PCR

RNA was extracted from the tissues using TRIzol (15596018, Thermo) according to the manufacturer’s instructions. Subsequently, PrimeScriptTMRT kit (R232-01, Vazyme) was used to generate cDNA. Real-time polymerase chain reaction (RT-PCR) was performed using SYBR Green Master Mix (Q111-02, Vazyme), and mRNA expression levels were normalized to the level of GAPDH mRNA. The expression levels were calculated using the 2^−ΔΔCt^ method. The primers used in this study were provided by Tsingke Biotech (Beijing, China) and are listed in **Supplementary Table S1**.

### 2.14 Cell counting kit-8 experiment

A cell suspension with a density of 3×10^3^ cells per well was seeded in 96-well plates. Subsequently, the plate was incubated in the dark at 37°C for 2 hours with 10 mL of CCK-8 labeling agent (A311-01, Vazyme) added to each well. The absorbance of the cells at 450 nm was measured using an enzyme-labeled meter (A33978, Thermo) at 0, 24, 48, 72, and 96 hours to determine cell viability.

### 2.15 Colony formation

1×10^3^ cells were transfected into each well of a 6-well plate and incubated for 14 days. The cells were then washed twice with PBS and fixed with 4% paraformaldehyde for 15 minutes prior to Crystal violet staining (Solarbio, China).

### 2.16 EdU

For the experiment, we seeded 2×10^4^ treated cells in each well of a 96-well plate after the cells had adhered to the wall. We then performed the 5-Ethynyl-2’-deoxyuridine (EdU) assay using the kit purchased from Ribobio (China). Finally, the number of proliferating cells was counted using an inverted microscope.

### 2.17 Wound-healing assay

Transfected cells were seeded in 6-well plates and cultured until they reached 95% confluency. A sterile 20-L plastic pipette tip was used to make a single straight scratch in each well, and unattached cells and debris were gently washed away with PBS. The width of the scratch wounds was measured using Image J software by taking photos at 0 and 48 hours.

### 2.18 Transwell assay

Transwell assays were used to investigate the migration and invasion ability of treated A549 and H1299 cells. Specifically, the top chamber of a 24-well plate was filled with 2×105 cells and incubated for 48 hours. To evaluate the invasion and migration capabilities, the top layer was either coated with matrigel solution (BD Biosciences, USA) or left untreated. After removing the cells on the top surface, the remaining cells on the bottom layer were fixed with 4% paraformaldehyde and stained with 0.1% crystal violet (Solarbio, China).

### 2.19 Animal models

Animal experiments were carried out in accordance with the guidelines of the Animal Experiment Ethics Committee of Nanjing Medical University. To establish a xenograft model, LGALS3-stably transfected H1299 cells and control cells were implanted into the left and right groin of 5-week-old BALB/c nude mice, respectively. Tumor size was measured every 5 days, and the xenograft tumors were harvested and weighed after 25 days.

### 2.20 Statistical analysis

Data processing, statistical analysis, and visualization were performed using R 4.2.0 software. The optimal cut-off value was determined using the “survminer” R package, and Kaplan-Meier analysis was conducted using the survival program (31, 35). The accuracy of the model was assessed using a ROC curve generated by the “timeROC” R package (36). For normally distributed variables, significant quantitative differences were identified using a two-tailed t-test or a one-way ANOVA, while for non-normally distributed data, a Wilcoxon test or a Kruskal Wallis test was used. Correlations between two continuous variables were assessed using Pearson’s correlation coefficients. A significance level of P < 0.05 was used.

## 3 Results

### 3.1 Analysis Process of scRNA-seq

**Figure 1:**
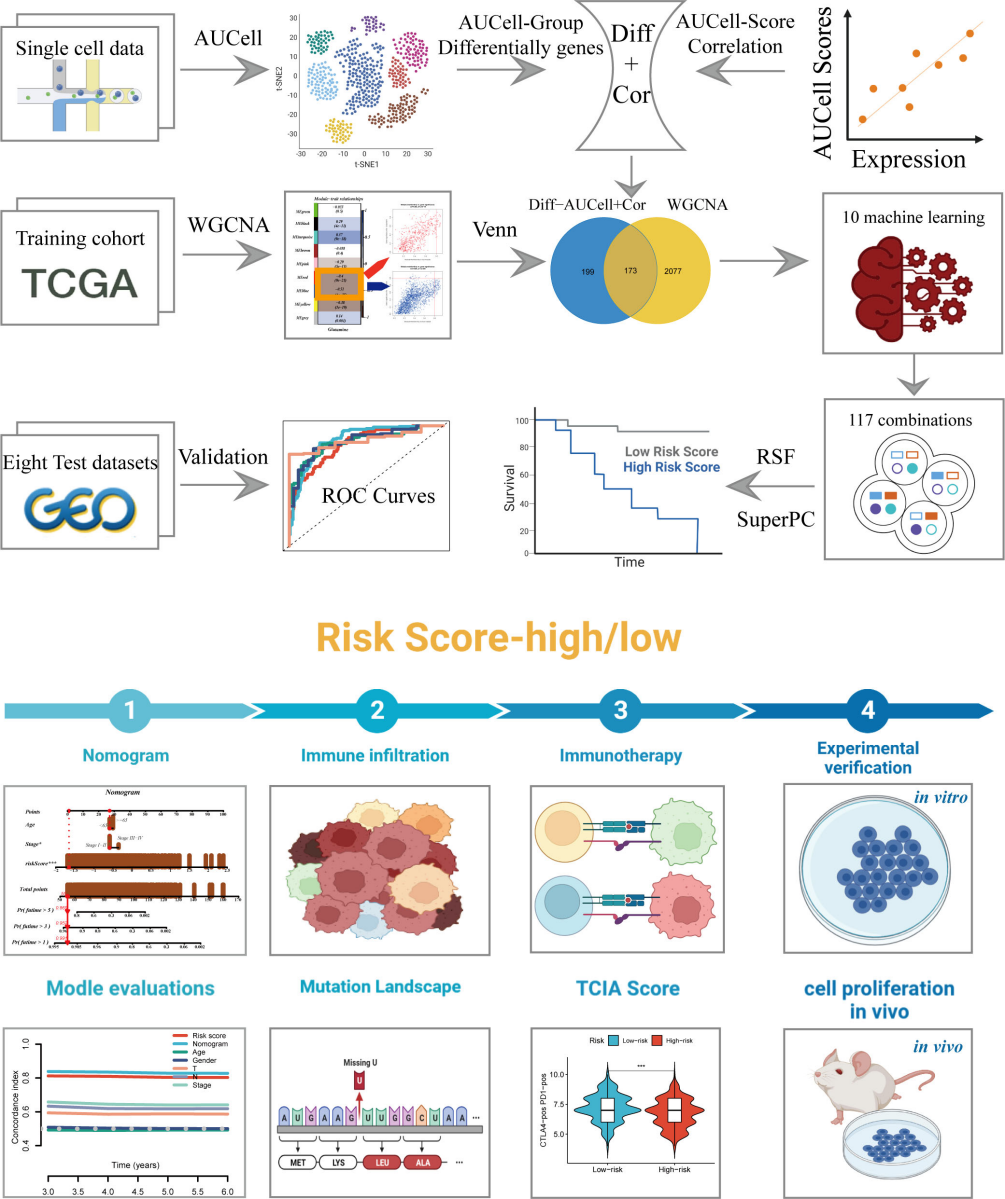
The workflow of the present study.

**Figure 2:**
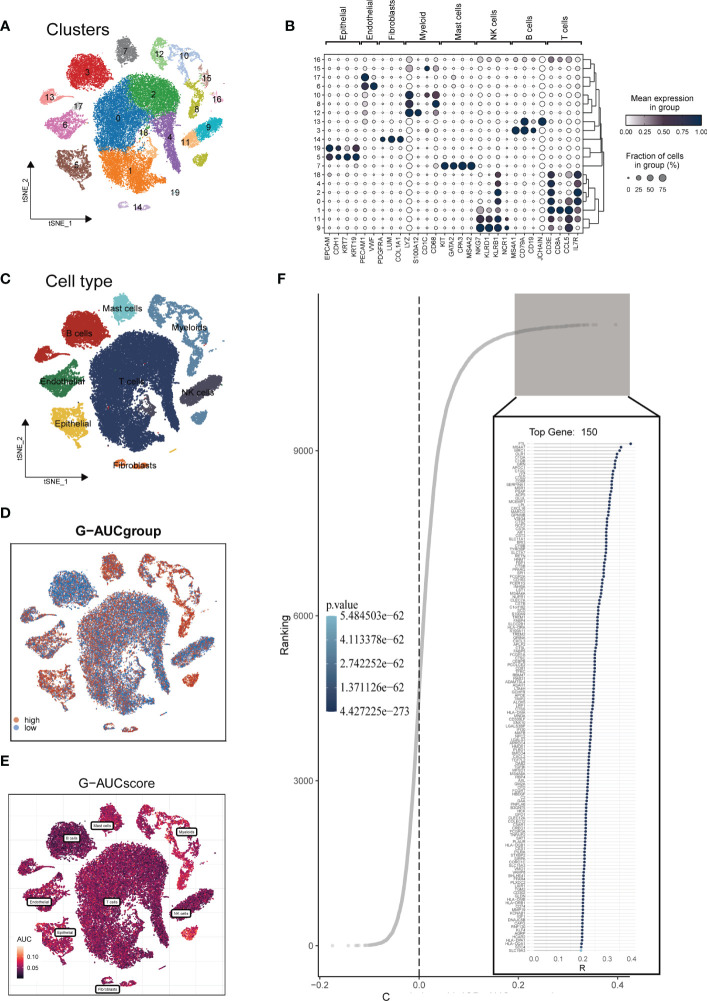
Single-cell data annotation. **(A)** The t-SNE plot revealed that all cells were classified into 20 distinct clusters. **(B)** A bubble plot was created to display the typical marker genes for each cell cluster. **(C)** The t-SNE map was used to identify 8 different cell types in the TME, as represented by different colors. **(D, E)** The AUCell score and groups of GM activity for each cell were visualized. **(F)** Correlation analysis was performed between the SM-AUCell score and genes.

The research process was illustrated in **Figure 1**. After filtering and quality control, 46,286 high-quality cells were retained for further analysis. **Supplementary Figure S1A** showed the expression patterns of each sample. The sequencing depth and total intracellular sequences had a significant positive correlation (R=0.94, **Supplementary Figure S1B**). The PCA plot revealed no apparent cell cycle changes **(** **Supplementary Figure S1C** **)**. The study included 12 samples, and the cell distribution within each sample was relatively consistent, indicating no notable batch effect across the samples, which could be used for future studies **(** **Supplementary Figure S1D** **)**. Next, dimensionality reduction techniques, including t-SNE, were used to classify all cells into 20 clusters **(** **Figure 2A** **)**. Bubble plots displayed the typical marker genes (21) of different cell types and their association with various clusters **(** **Figure 2B** **)**. We presented a t-SNE plot to show the distribution of each cell population **(** **Figure 2C** **)**. We evaluated the GM activity of each cell, and cells that expressed more glutamine metabolism-related genes (GMRGs), mainly orange-colored myeloid cells, exhibited higher AUC values **(** **Figure 2D** **)**. We assigned an AUC score for the GMRGs to all cells and classified them into high- and low-GM-AUC groups based on the median AUC score **(** **Figure 2E** **)**. Subsequently, we performed differential analysis to identify DEGs associated with glutamine metabolism in the high- and low-GM-AUC groups. Moreover, we conducted correlation analysis to investigate the most closely associated genes with GM activity **(** **Figure 2F** **)**, and the top 150 genes with the highest correlation coefficients were included in further research. The DEGs and the genes obtained from the correlation analysis were integrated into single-cell analysis to identify the genes that had the most significant impact on GM activity (a total of 449 genes).

### 3.2 Identification of the most relevant genes for GM activity

**Figure 3:**
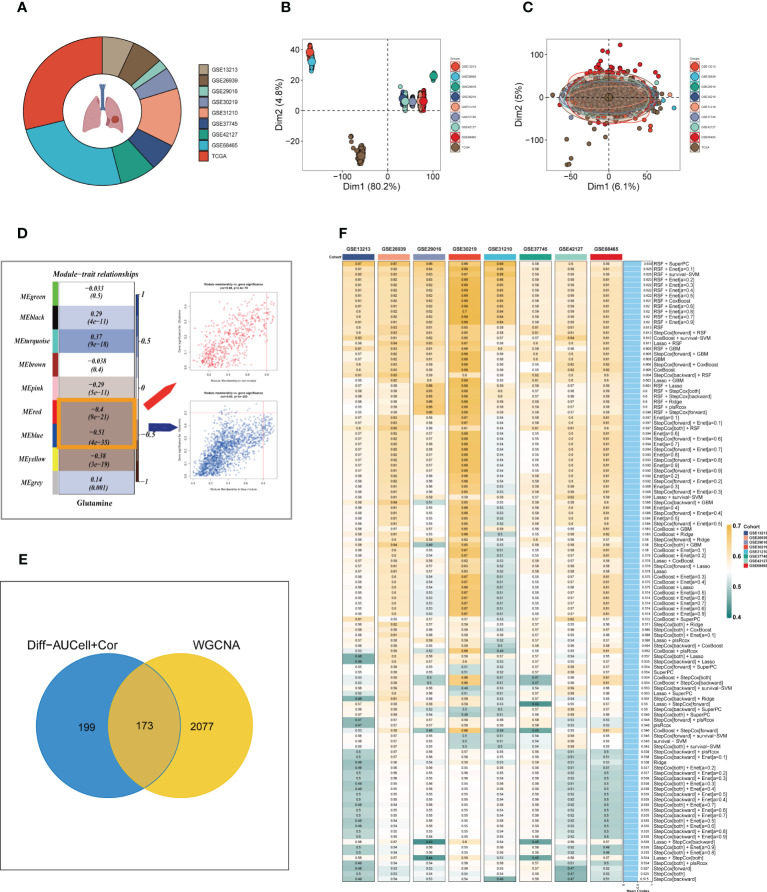
Construction of the GMAS. **(A)** The sources of samples and the proportion of sample size in 10 datasets were analyzed. **(B, C)** PCA plots before and after removal of batch effects for 10 datasets. **(D)** WGCNA analysis searched for the modules most associated with GM activity. **(E)** Venn plots identified the genes most associated with GM activity. **(F)** A total of 117 kinds of prediction models via LOOCV framework and further calculated the C-index of each model across all validation datasets.

To enhance data consistency, we removed the batch effect from both the GEO-obtained data and the TCGA data. **Figure 3A** depicts the distribution ratio of the nine data sets, while **Figures 3B, C** shows the PCA plots before and after removing the batch effect, respectively. We obtained the GM score of each TCGA-LUAD sample using ssGSEA and searched for gene sets covariant with the GM score using WGCNA. **Supplementary Figure S2A** indicates that the data tends to be more consistent with the power-law distribution when the soft domain value is set to 7. Furthermore, when the minimum number of modules was set to 100, deepSplit to 3, and the modules with similarity less than 0.25 were merged **(** **Supplementary Figure S2B** **)**, nine non-gray modules were generated **(** **Figure 3D** **)**. We examined the relationship between the expression of each module and clinical features. Finally, the red and blue modules were identified as the most relevant to GM. We intersected the 449 genes most associated with GM activity identified in the scRNA-seq analysis with the two module genes most associated with GM activity identified in WGCNA, resulting in a total of 173 overlapping genes for further analysis **(** **Figure 3E** **)**.

### 3.3 Building a consensus signature

We utilized a machine learning-based integrative approach to establish a consensus GM-associated signature (GMAS) using the 173 overlapping genes. We employed the LOOCV framework to evaluate the performance of the GMAS by fitting 117 different prediction models to the TCGA-LUAD dataset and assessing the C-index of each model across all validation datasets **(** **Figure 3F** **)**. Notably, the best model with the highest average C-index (0.639) was a combination of RSF and superPC, and this combined model demonstrated the best C-index in all validation datasets. Using the RSF algorithm, we identified 22 genes with high importance, and based on these genes, the SuperPC algorithm calculated a risk score for each sample.

### 3.4 Survival analysis and model evaluation

**Figure 4:**
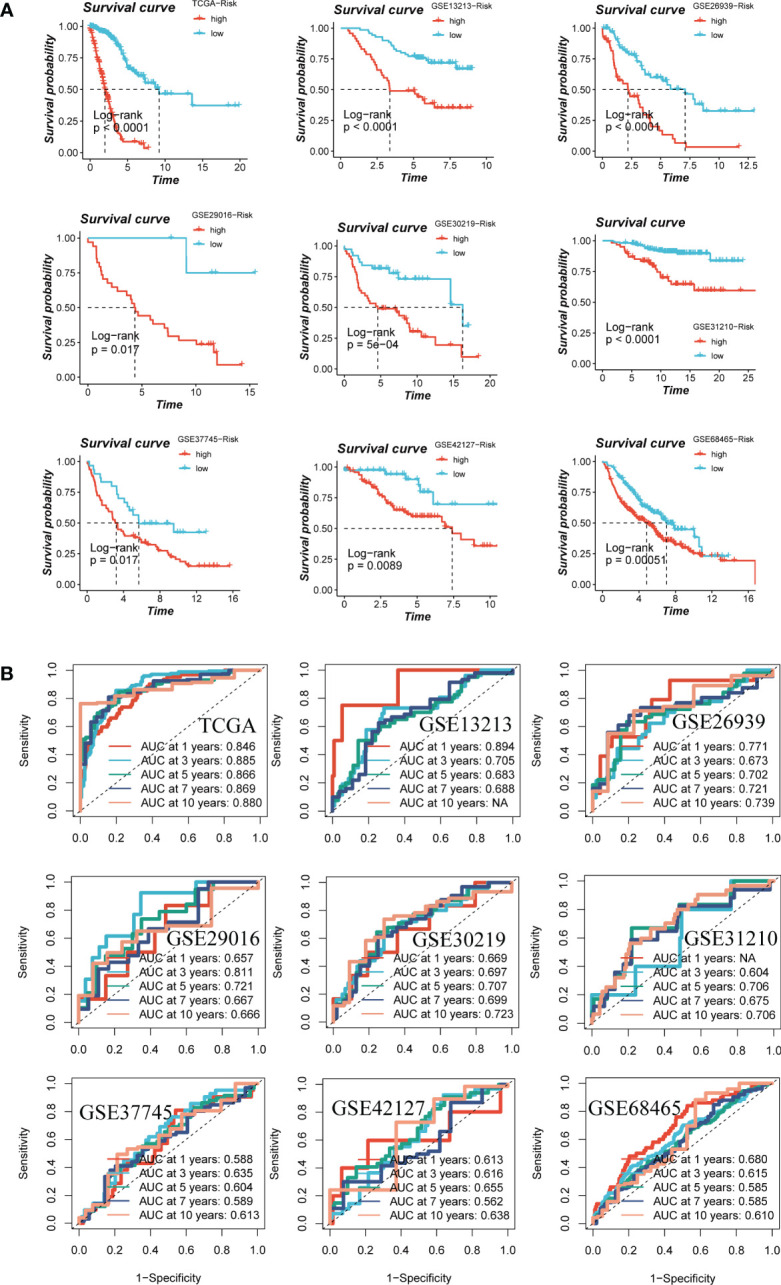
Assessment of risk models. **(A)** Kaplan-Meier survival analysis of signatures in the TCGA and eight GEO datasets. **(B)** The ROC curve was used to evaluate the performance of the model in the TCGA and eight GEO datasets.

We categorized patients into high- and low-risk groups based on their median risk values and observed significant differences in overall survival (OS) for TCGA-LUAD patients as well as in eight GEO datasets, as depicted in **Figure 4A**. To assess the discriminative ability of the GMAS, ROC analysis was performed, yielding AUCs of 0.846, 0.885, 0.866, 0.869, and 0.880 for TCGA-LUAD at 1-, 3-, 5-, 7-, and 10-year time points, respectively. The AUCs for GSE13213 (lacking LUAD patients with survival > 10 years) were 0.894, 0.705, 0.683, and 0.688, while for GSE26939, the AUCs were 0.771, 0.673, 0.702, 0.721, and 0.739. In the case of GSE29016, the AUCs were 0.657, 0.811, 0.721, 0.667, and 0.666, and for GSE30219, the AUCs were 0.669, 0.697, 0.707, 0.699, and 0.723. For GSE31210 (lacking LUAD patients with survival < 1 year), the AUCs were 0.604, 0.706, 0.675, and 0.706, and for GSE37745, the AUCs were 0.588, 0.635, 0.604, 0.589, and 0.613. Finally, for GSE42127, the AUCs were 0.613, 0.616, 0.655, 0.562, and 0.638, while for GSE37745, the AUCs were 0.680, 0.615, 0.585, 0.585, and 0.610, respectively, as presented in **Figure 4B**.

### 3.5 Construction and validation of prognostic nomogram

**Figure 5:**
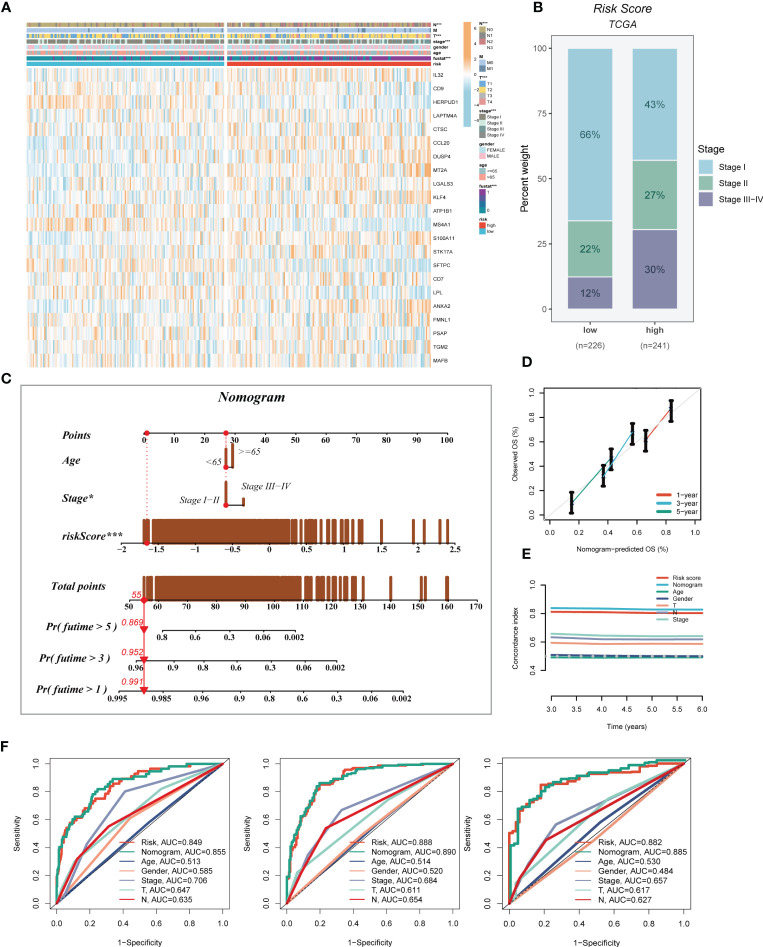
Developing an accurate nomogram. **(A)** A heatmap was generated to integrate clinical data with the expression of model genes. **(B)** The proportion of clinical stage was visualized in different risk groups. **(C)** The nomogram was constructed by combining clinical features with risk score. **(D)** Calibration plots were used to assess the consistency between actual OS rates and predicted survival rates. The 45° line represents the best possible prediction. **(E)** C-index curves were utilized to evaluate the predictive performance of different clinical characteristics, nomogram scores, and risk scores. **(F)** ROC curves were generated for 1, 3, and 5 years to illustrate AUC values for various clinical factors, risk scores, and nomogram scores. *P < 0.05, ***P < 0.001.

A heatmap was generated to display the relationship between the model genes and clinical features. Significant differences (P < 0.001) were observed between the high- and low-risk groups in terms of clinical parameters such as T stage, N stage, clinical stage, and survival status **(** **Figure 5A** **)**. Furthermore, we compared the distribution of different stages among the groups and presented it as a percentage bar plot. The high-risk group was found to have a higher proportion of clinical stage II and III-IV patients, whereas stage I patients dominated the low-risk group **(** **Figure 5B** **)**. We developed a predictive nomogram that incorporated the risk score and clinicopathological factors (age and clinical stage) based on the TCGA-LUAD dataset to better predict prognosis **(** **Figure 5C** **)**. Clinical outcomes, such as survival status at 1, 3, and 5 years, were used as parameters. The calibration plot indicated that the GMAS had excellent predictive performance for 1-, 3-, and 5-year survival rates **(** **Figure 5D** **)**. The C-index curves indicated that the nomogram predicted prognosis better than the risk score and any other clinical parameter **(** **Figure 5E** **)**. We also performed ROC analysis to evaluate the predictive performance of the nomogram score, risk score, and other clinical features. The AUC values of the nomogram score at 1, 3, and 5 years were 0.855, 0.890, and 0.885, respectively, which were higher than those of the risk scores and other clinical parameters **(** **Figure 5F** **)**.

### 3.6 Differences in the immune microenvironment and immunotherapy response

**Figure 6:**
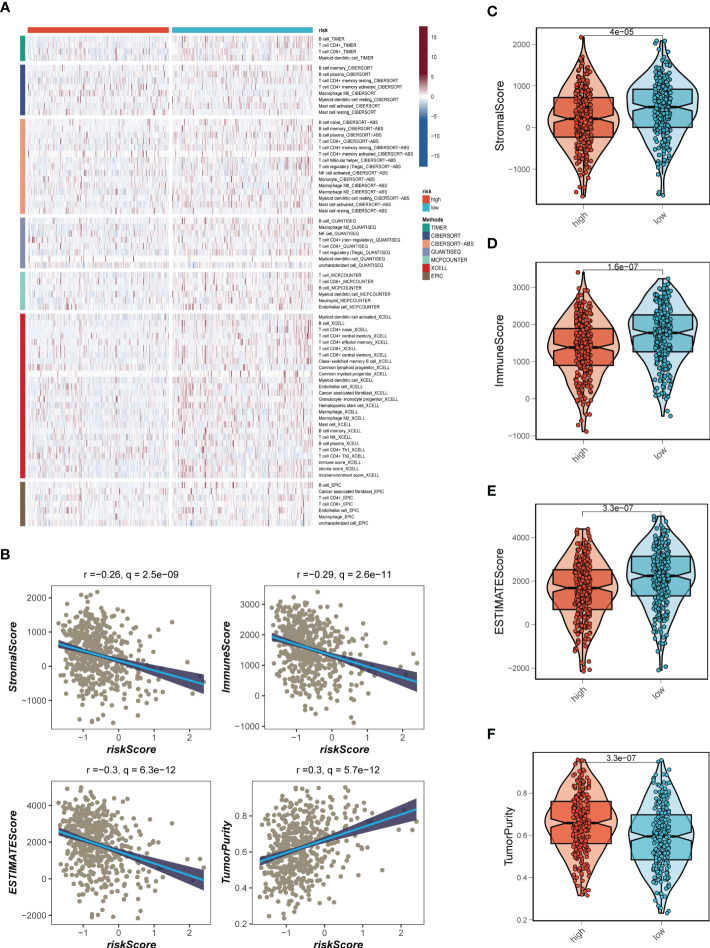
Analysis of immune infiltration. **(A)** Seven algorithms assess differences in immune infiltration status between different risk groups. **(B)** The correlations in Stromal Score, Immune Score, ESTIMATE Score, and tumor purity calculated using the ESTIMATE algorithm between the two risk subgroups. **(C-F)** The violin plot demonstrated the difference in Stromal Score, Immune Score, ESTIMATE Score, and tumor purity calculated using the ESTIMATE algorithm between the two risk subgroups.

**Figure 7:**
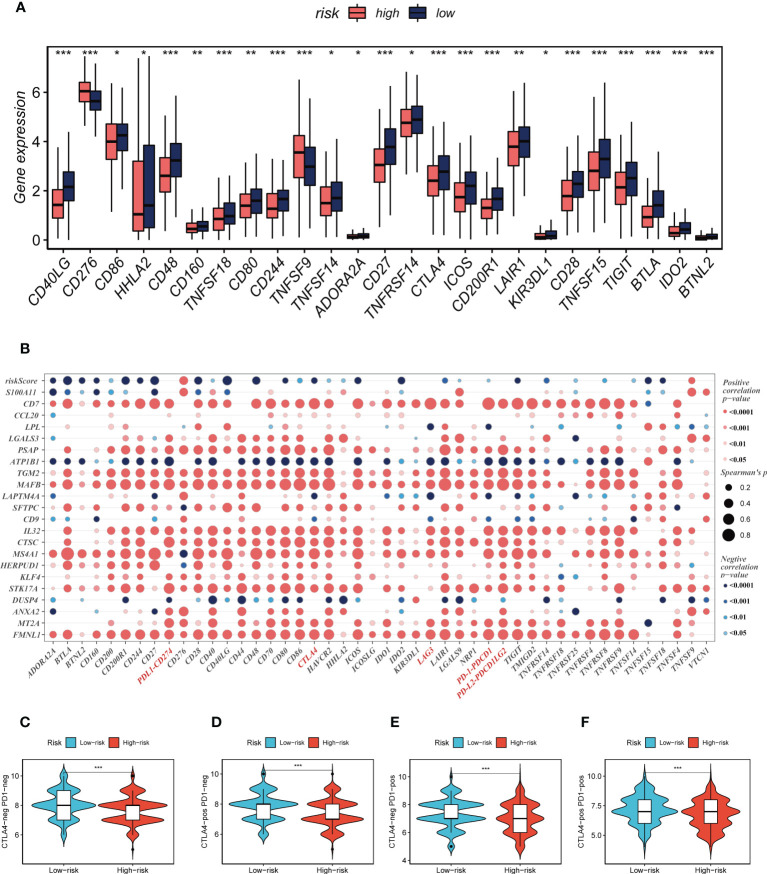
Immune checkpoint and TCIA analysis. **(A)** A box plot showed that differences in immune checkpoint gene expression between high- and low-risk groups. **(B)** Correlation between model genes and immune checkpoint. **(C-F)** The low-risk group has significantly greater IPS, IPS-CTLA4-neg-PD-1-neg, IPS-CTLA4-pos-PD-1-neg, IPS-CTLA4-neg-PD-1-pos, and IPS-CTLA4-pos-PD-1-pos. *P < 0.05, **P < 0.01, ***P < 0.001.

Seven different algorithms were employed to demonstrate that high-risk tumors had more infiltration of immune cells such as T cells, B cells, NK cells, and activated mast cells, as depicted in **Figure 6A**. We utilized the ESTIMATE method to assess the level of immune infiltration in different risk groups. Spearman correlation analysis was conducted to explore the association between the risk score and immune infiltration score. The risk scores were found to be significantly and negatively correlated with stromal (R = -0.26, FDR < 0.001), immune (R = -0.29, FDR < 0.001), and ESTIMATE scores (R = -0.30, FDR < 0.001), while positively correlated with tumor purity (R = 0.30, FDR < 0.001, **Figure 6B**). Likewise, **Figures 6C–F** confirmed the earlier findings, with the low-risk group displaying higher stromal, immune, and ESTIMATE scores (combined stromal and immune score). According to the results, the risk score was associated with the level of immune cell infiltration and the amount of each component in the tumor microenvironment. Different levels of immune infiltration can lead to varying disease progression and effectiveness of immunotherapy. Based on the above findings, we investigated whether the prognostic model could predict the response of LUAD patients to immune checkpoint inhibitors (ICIs). We first analyzed the relationship between the risk score and well-established immunotherapy biomarkers in the TCGA-LUAD cohort. The analysis revealed that almost all ICGs, such as CD40LG, TIGIT, and CTLA4, were highly expressed in the high-risk group **(** **Figure 7A** **)**. Subsequently, the correlations between modeling genes, risk scores, and ICGs were examined and shown in the bubble plot **(** **Figure 7B** **)**, with blue and orange representing negative and positive correlations, respectively, with larger bubbles and darker colors indicating a higher degree of association. The IPS has been utilized to identify individuals who may be highly responsive to immunotherapy. Based on this score, tumor samples were evaluated to determine if they would exhibit a favorable immune response to either PD-1/PD-L1 or CTLA4 inhibitors, or both (as illustrated in **Figures 7C–F**). Notably, patients classified in the low-risk group demonstrated significantly higher IPS scores, indicating that they may derive the greatest benefit from this type of immunotherapy. These findings hold important clinical implications and suggest that the IPS score could serve as a useful tool in the identification and stratification of patients who are most likely to benefit from immunotherapy.

### 3.7 Mutational landscape

**Figure 8:**
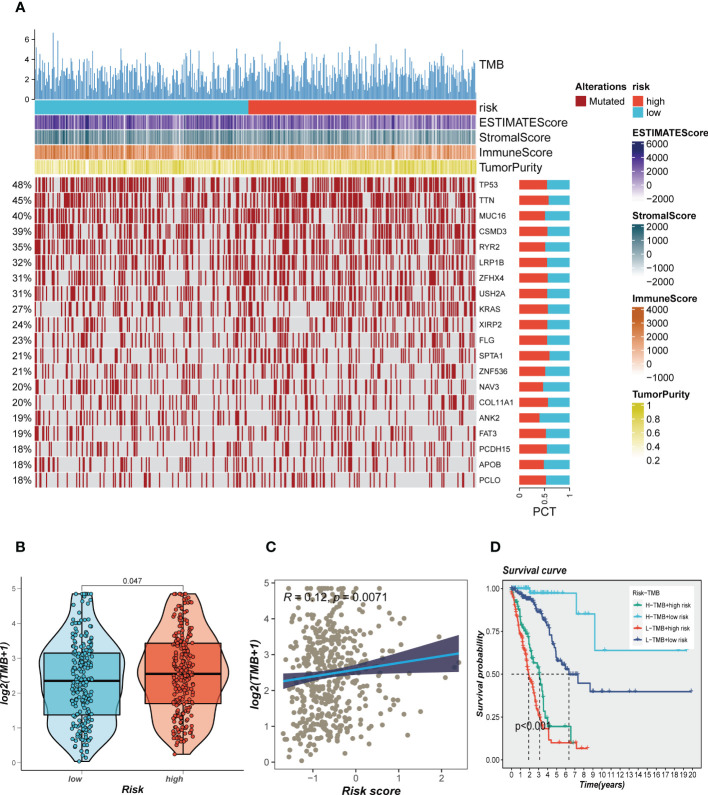
Landscape of LUAD sample mutation profiles. **(A)** Mutation landscape of the top 20 genes with mutation frequency in differential risk subgroups. **(B)** Comparison of tumor mutation burden (TMB) between different risk groups. **(C)** Correlation analysis between risk score and TMB. **(D)** Survival differences for four different subgroups (H-TMB+high-risk, H-TMB+low-risk, L-TMB+high-risk, and L-TMB+low-risk).

Genetic mutations are crucial in personalized cancer treatment. Therefore, we analyzed somatic mutation profiles of different risk groups. The top 20 frequently mutated genes, such as TP53, TTN, and CSMD3, had a higher mutation frequency in the high-risk group, as shown in **Figure 8A**. In addition, there was a significant difference in tumor mutation burden (TMB) between high- and low-risk groups, with higher TMB in the high-risk group **(** **Figure 8B** **)**. Spearman correlation analysis revealed a positive correlation between risk score and TMB (R = 0.12, P < 0.001, **Figure 8C**). We further divided patients into four groups based on the median TMB and median risk values. LUAD patients in the H-TMB+low-risk group had the best prognosis, while those in the L-TMB+high-risk group had the worst prognosis, as shown in **Figure 8D**.

### 3.8 Functional enrichment analysis

**Figure 9:**
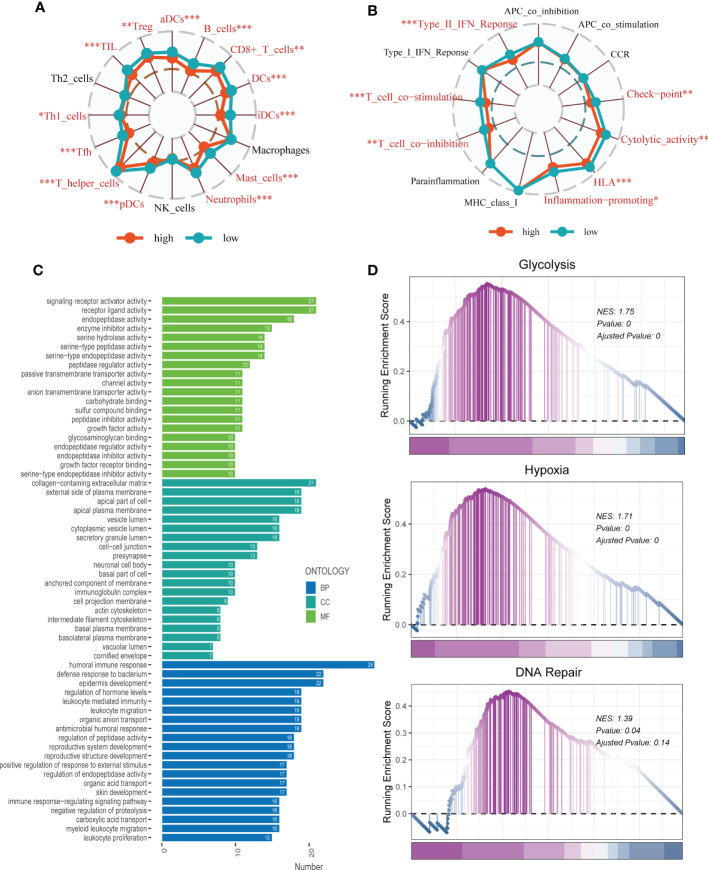
Enrichment analysis. **(A, B)** The ssGSEA algorithm was employed to quantify the immune cell infiltration and immune function between the high-risk and low-risk groups. *P < 0.05, **P < 0.01, ***P < 0.001; ns, not significant, p value > 0.05. **(C)** A bar plot showed GO enrichment analysis. **(D)** GSEA showed pathway differences between high- and low-risk groups.

To investigate immune status variations across different risk groups, we used the ssGSEA algorithm. Low-risk LUAD patients showed increased infiltration of various immune cells, including aDCs, B cells, CD8+ T cells, DCs, iDCs, Mast cells, neutrophils, pDCs, Th1/Th2 cells, TILs, and Tregs. Moreover, the low-risk group exhibited significantly higher levels of Checkpoints, Cytolytic activity, HLA, Inflammation promoting, T cell co-inhibition, T cell co-stimulation, and Type II IFN response compared to the high-risk group **(** **Figures 9A, B** **)**. Differential expression analysis revealed genes that were differentially expressed in both high- and low-risk groups (P < 0.05 and log2 (FC) > 1). Subsequently, the DEGs were subjected to GO enrichment analysis **(** **Figure 9C** **)**, which showed that the top three enriched pathways in Biological Processes (BP) were signaling receptor activator activity, receptor-ligand activity, and endopeptidase activity; Cellular Component (CC) included collagen-containing extracellular matrix, external side of the plasma membrane, and apical part of the cell; and Molecular Function (MF) involved humoral immune response, defense response to the bacterium, and epidermis development. Additionally, GSEA enrichment analysis revealed that DNA Repair (NES = 1.39, p = 0.04), Glycolysis (NES = 1.75, p = 0.000), and Hypoxia (NES = 1.71, p = 0.000) were primarily enriched in the high-risk group **(** **Figure 9D** **)**.

### 3.9 Experimental verification

**Figure 10:**
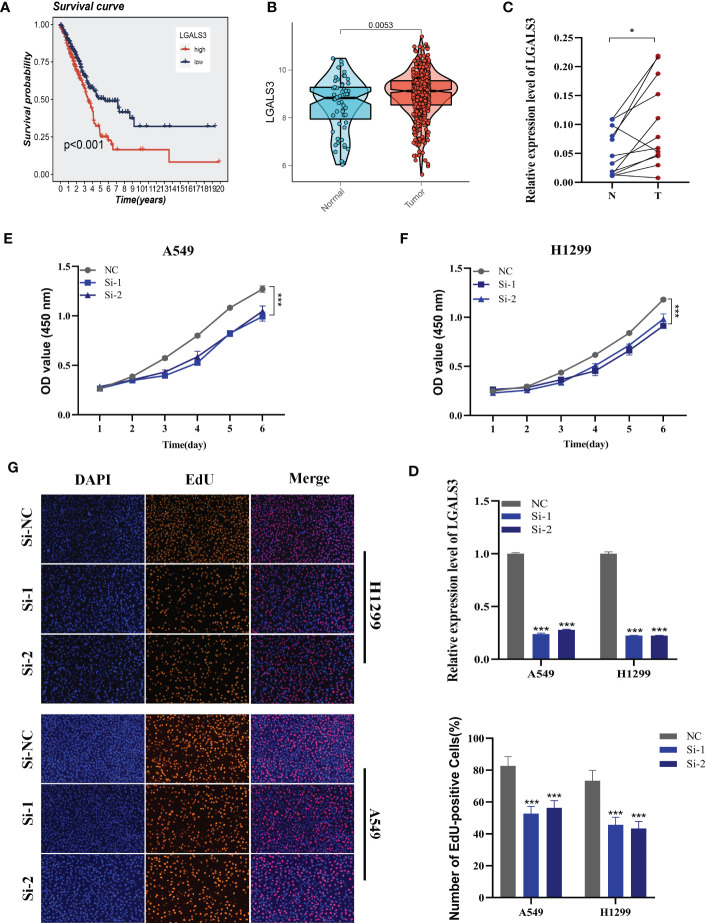
Cell experiment. **(A)** Survival analysis showed the effect of LGALS3 expression on prognosis. **(B)** The difference in LGALS3 expression between normal samples and tumor samples was found in the TCGA database. **(C)** Relative expression of LGALS3 in tumor and paracancerous tissues in LUAD and LGALS3 was highly expressed in tumor tissues compared with adjacent tissues **(D)** qRT-PCR to evaluate the level of LGALS3 expression 5 days after transfection and siRNA sequences could result in a significant decrease in LGALS3 expression (P < 0.001). **(E, F)** CCK8 assay showed that, after LGALS3 knockdown, the cells showed a significant reduction in viability. **(G)** EdU staining assay indicated that downregulation of LGALS3 expression repressed cell proliferation in LUAD cell lines. *P < 0.05, ***P < 0.001.

**Figure 11:**
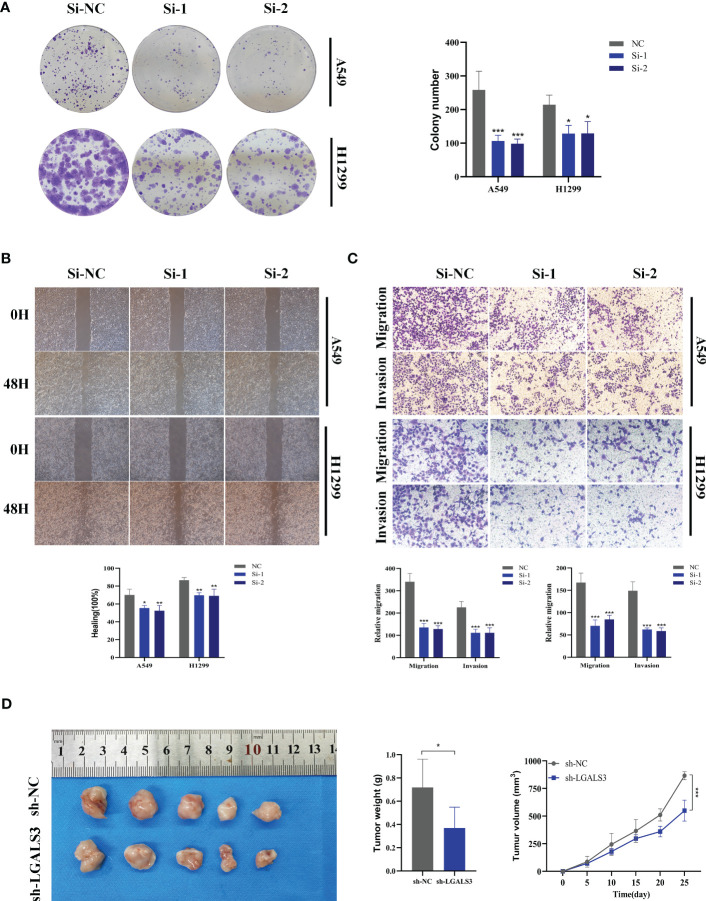
Xenograft tumor in Nude Mice. **(A)** Colony formation assay displayed that cell with reduced LGALS3 expression exhibited a significant reduction in the numbers of colonies, compared with the NC group. **(B)** Scratch-wound healing assay depicted that a significantly slower wound healing rate was observed in cells with a decreased expression of LGALS3. **(C)** Transwell assay showed that downregulation of LGALS3 expression inhibited the migration and invasion capacity of LUAD cells. **(D)** Nude mice experiments. LGALS3 knockdown inhibited tumor growth, and tumor volume and weight were smaller than those in the control group. To demonstrate the accuracy and reproducibility of the results, all experiments were repeated in two LUAD (A549, H1299) cell lines and all data were presented as the means ± SD of three independent experiments. *P < 0.05, **P < 0.01, ***P < 0.001.

Using the String database (37), we constructed a protein interaction network plot **(** **Supplementary Figure S3A** **)**, which showed that LGALS3 was highly connected to other model genes, indicating its importance as a core gene in the network. LGALS3 was found to be highly expressed in tumors **(** **Figures 10A, B** **)**, and its overexpression was associated with worse prognosis in LUAD patients in the TCGA database. The prognostic significance of LGALS3 was also confirmed using the GSE31210 dataset **(** **Supplementary Figure S4A** **)**. Further GSVA enrichment analysis revealed that patients with high LGALS3 expression were significantly enriched in P53 pathway, interferon alpha response and apoptosis pathway **(** **Supplementary Figure S5** **)**. To further validate these findings, we conducted functional experiments both *in vitro* and *in vivo*. Firstly, we verified the expression levels of LGALS3 in LUAD and adjacent non-tumor samples and found that LGALS3 was highly expressed in LUAD samples **(** **Figure 10C** **)**. Next, we assessed the efficiency of siRNA knockdown of LGALS3 in A549 and H1299 cell lines using qRT-PCR **(** **Figure 10D** **)**. CCK-8 and EdU assays showed that knockdown of LGALS3 led to decreased proliferation of A549 and H1299 cells compared to the control group **(** **Figures 10E–G** **)**, suggesting that LGALS3 plays a role in promoting the proliferation of LUAD cell lines. Clonogenic assays demonstrated that knockdown of LGALS3 reduced the ability of LUAD cells to form colonies **(** **Figure 11A** **)**, while wound healing and transwell assays showed that LGALS3 knockdown significantly inhibited the migration and invasion of LUAD cells **(** **Figures 11B, C** **)**. Finally, *in vivo* experiments showed that LGALS3 knockdown suppressed tumor growth, with smaller tumor volume and weight compared to the control group **(** **Figure 11D** **)**, suggesting that LGALS3 functions as a pro-oncogenic regulator in LUAD tumorigenesis and progression.

## 4 Discussion

Despite advancements in diagnostic methods and treatment protocols, lung cancer (LC) still accounts for the highest cancer-related morbidity and mortality globally (38). In the case of early-stage LUAD patients, surgery is commonly recommended. However, for those with advanced LUAD, a combination of chemotherapy, radiotherapy, immunotherapy, targeted therapy, or a combination of these treatments may be more effective (39). Studies have shown that immunotherapy is prone to immune tolerance and is not always successful, hence, there is an urgent need to explore new immune targets.

Glutamine, a non-essential amino acid, plays a crucial role in tumorigenesis and the tumor microenvironment (TME). Various therapeutic targets and specific blockers targeting metabolic dysregulation have been reported (40). However, inhibiting a single metabolic target is often inadequate to restrain cancer growth in preclinical trials. Therefore, novel targets and synergistic therapies are promising. In this study, we investigated a novel diagnostic signature of GMRGs and immunotherapy targets, which provides prospects for reversing immune resistance and improving the prognosis of patients.

In this study, single-cell RNA sequencing was utilized to evaluate 12 LUAD samples, which allowed identification of eight distinct cell types. By using the AUCell method and GM gene set retrieved from GeneCards, we discovered that myeloid cells exhibited the highest levels of GM activity, which implies that GM may have a crucial role in regulating carcinogenesis and development through the modulation of myeloid cells. Key genes that regulate GM activity were identified, and an integrative workflow was developed to create a consensus GMAS using the expression profiles of 173 such genes. To generate the GMAS, 117 different models were fit to the training dataset using the LOOCV framework. The RSF and SuperPC algorithms were found to produce the best results. Our prognostic analysis indicated that the high-risk group had a worse prognosis. Additionally, ROC analysis showed that the GMAS had high accuracy and consistent performance across eight public GEO datasets. To enhance the predictive power of our analysis, we incorporated clinical information and created a nomogram. Our findings demonstrated that the nomogram scores had a better predictive performance for survival compared to risk scores and other clinical characteristics.

The tumor microenvironment (TME) comprises diverse components, including the extracellular matrix, cancer-associated fibroblasts, new blood vessels, endothelial cells, and tumor-infiltrating immune cells. These components can have either positive or negative impacts on tumor prognosis, depending on their roles in promoting tumor destruction, increasing tumor invasiveness, or enhancing anti-therapeutic response (41). In this study, we evaluated immune cell infiltration in high- and low-risk LUAD patients to understand how the TME influences tumor prognosis. Seven different algorithms were used to measure immune cell infiltration in different risk groups, and the results showed that tumors in the low-risk group had higher levels of immune cell infiltration. Using the ESTIMATE method, we also found that low-risk samples had higher immune cell infiltration, and the risk score was negatively correlated with stromal, immune, and ESTIMATE scores (FDR < 0.001). Furthermore, we observed that most of the known immune checkpoint genes were highly expressed in the low-risk group, and correlation analysis indicated that risk scores were significantly negatively correlated with most of the immune checkpoint genes. To assess the variances in immunotherapy efficacy in different risk groups, we explored the effects of PD-1 and CTLA-4 treatment using TCIA. The findings suggested that LUAD patients in the low-risk group were likely to benefit more from immunotherapy since their IPS score was significantly higher than that of the high-risk group.

Recent studies have highlighted the connection between genetic alterations, neoantigen production, and immunotherapeutic response (42). Surprisingly, our findings showed that patients in the low-risk group had lower TMB levels, while those in the high-risk group had a greater frequency of mutations in high-risk genes. Based on the median TMB values and median risk values, we divided the patients into four categories: H-TMB+high-risk, H-TMB+low-risk, L-TMB+high-risk, and L-TMB+low-risk. The H-TMB+low-risk group exhibited the most favorable prognosis, which may provide new recommendations for the clinical assessment of patient outcomes.

Various pathway enrichment analyses were conducted to unravel the underlying mechanisms responsible for survival disparities between the two risk groups. GSEA analysis revealed that pathways involved in DNA repair, glycolysis, and hypoxia were predominantly enriched in the high-risk group. The DNA repair pathway is essential in maintaining genomic stability, and its defects may contribute to tumorigenesis (43). Glycolysis is a unique metabolic pathway that takes place mainly in the cytoplasm and does not require oxygen molecules. It produces ATP, which has become the primary source of energy for the growth and metabolism of cancer cells. However, oxygen is a crucial energy metabolite that drives cellular biological functions. The rapid and uncontrolled proliferation of tumors results in a limited availability of oxygen, leading to hypoxia, a common microenvironmental feature in almost all solid tumors. Hypoxia has been considered a promising therapeutic target (44).

By analyzing the String database through protein-protein interaction analysis, LGALS3 was found to be a core gene in the gene network. In the TCGA database, LGALS3 was highly expressed in tumor groups, and high expression of LGALS3 was associated with poor prognosis in LUAD patients. To further investigate the underlying mechanism, we conducted several experiments and found that knockdown of LGALS3 significantly reduced the invasion, migration, and proliferation of LUAD cell lines. However, it should be noted that further research is necessary to validate these findings and explore the potential therapeutic implications of targeting LGALS3 in LUAD.

It is important to note some limitations of this study. Firstly, the prognostic signature was developed based on existing datasets and requires validation in larger prospective clinical trials. Furthermore, although our results indicate that the signature could potentially serve as a prognostic biomarker and predictor of immunotherapy response, additional research is needed to verify these results. Nevertheless, our integrated analysis utilizing machine learning provides valuable insight into the prognostic significance and potential therapeutic implications of GMRGs in LUAD.

## Data availability statement

The original contributions presented in the study are included in the article/**Supplementary Material**. Further inquiries can be directed to the corresponding authors.

## Ethics statement

The human study was approved by Medical Ethics Committee (2019-SR-156). Animal experiments were carried out in accordance with the guidelines of the Animal Experiment Ethics Committee of Nanjing Medical University.

## Author contributions

PZ, SP, and LW contributed conception and design of the study. ZX, QW, and XH collected the data. ZL and JX performed the statistical analysis. PZ, SP, and LW wrote the first draft of the manuscript. MD and HL gave the final approval of the version to be submitted. All authors contributed to manuscript and approved the submitted version
