# Supplementary material for: Integrating multiple machine learning methods to construct glutamine metabolism-related signatures in lung adenocarcinoma
Source: Front Endocrinol (Lausanne). 2023 May 17;14:1196372. doi: 10.3389/fendo.2023.1196372 (PMC10229769; doi:10.3389/fendo.2023.1196372)
Supplement: Supplementary file 1 [file Image_1.pdf]

Supplementary Material

Supplementary Figures

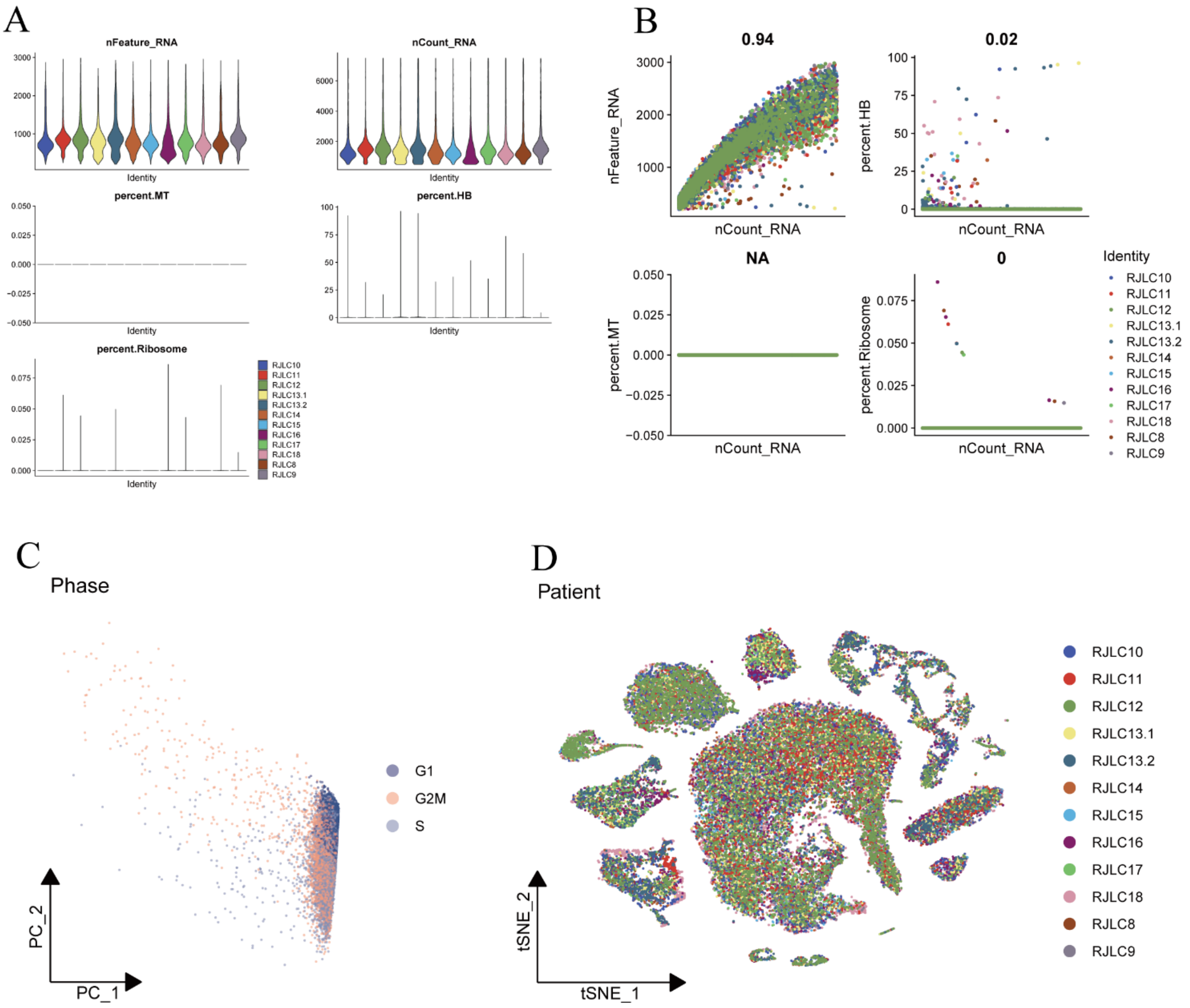

Supplementary Figure S1. Quality control of single-cell sequencing data and model construction. (A) The distribution of gene expression levels, sequencing depth, the percentage of red blood cell genes, the percentage of mitochondrial genes, and the percentage of ribosome genes in the 12 samples. (B) Correlation between sequencing depth and gene expression levels, the percentage of mitochondrial genes, the percentage of red blood cell genes, and the percentage of ribosome genes.

(C) Cell cycle-related genes were used for dimensionality reduction clustering. (D) The t-SNE plot showed all the cells in the 12 LUAD samples.

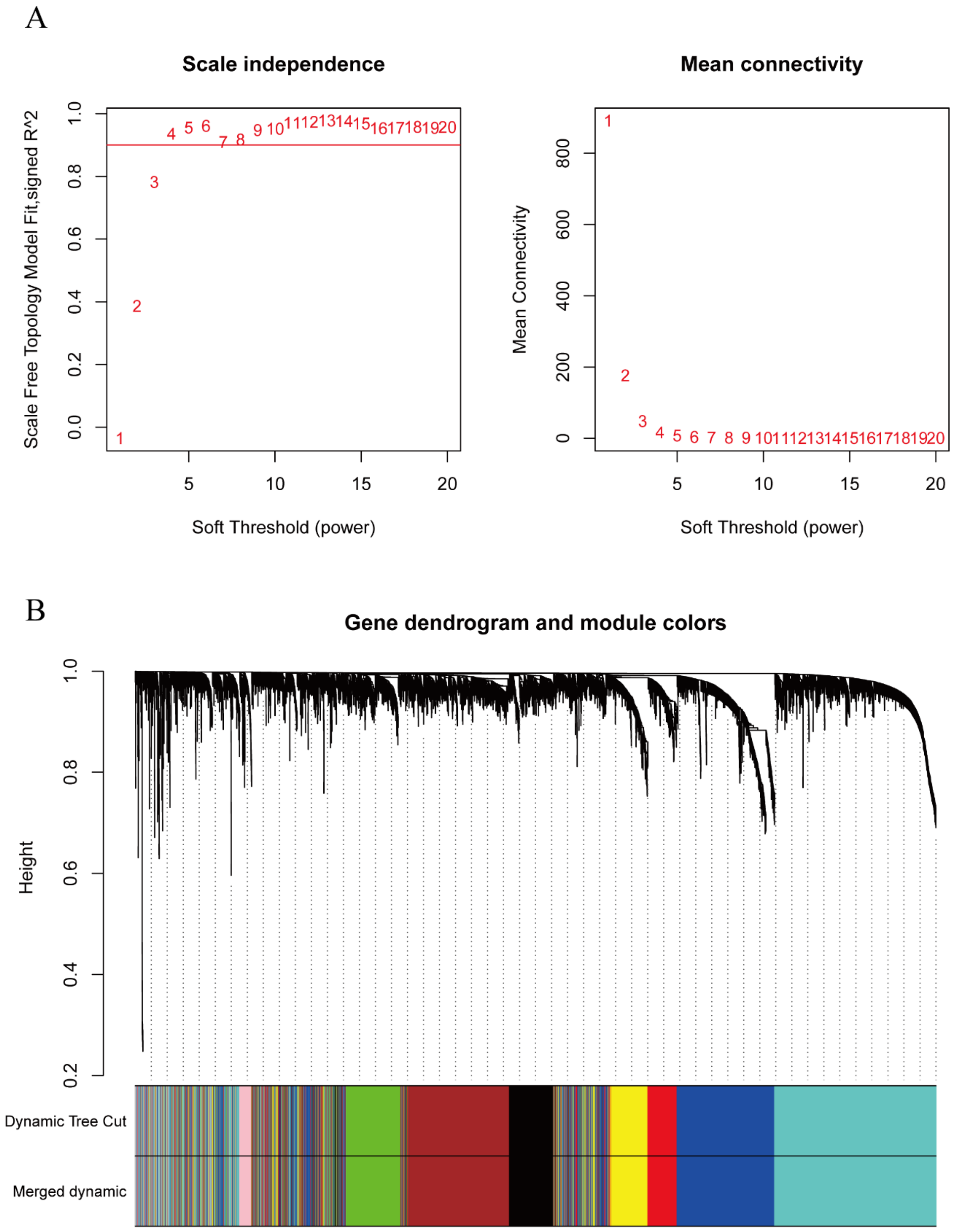

Supplementary Figure S2. WGCNA analysis. (A) The soft domain value is 7, the data is more consistent with the power-law distribution, and mean connectivity tends to be stable. (B) The

minimum number of modules was set to 100 and deepSplit to 3 and merging the modules with a similarity lower than 0.25.

A

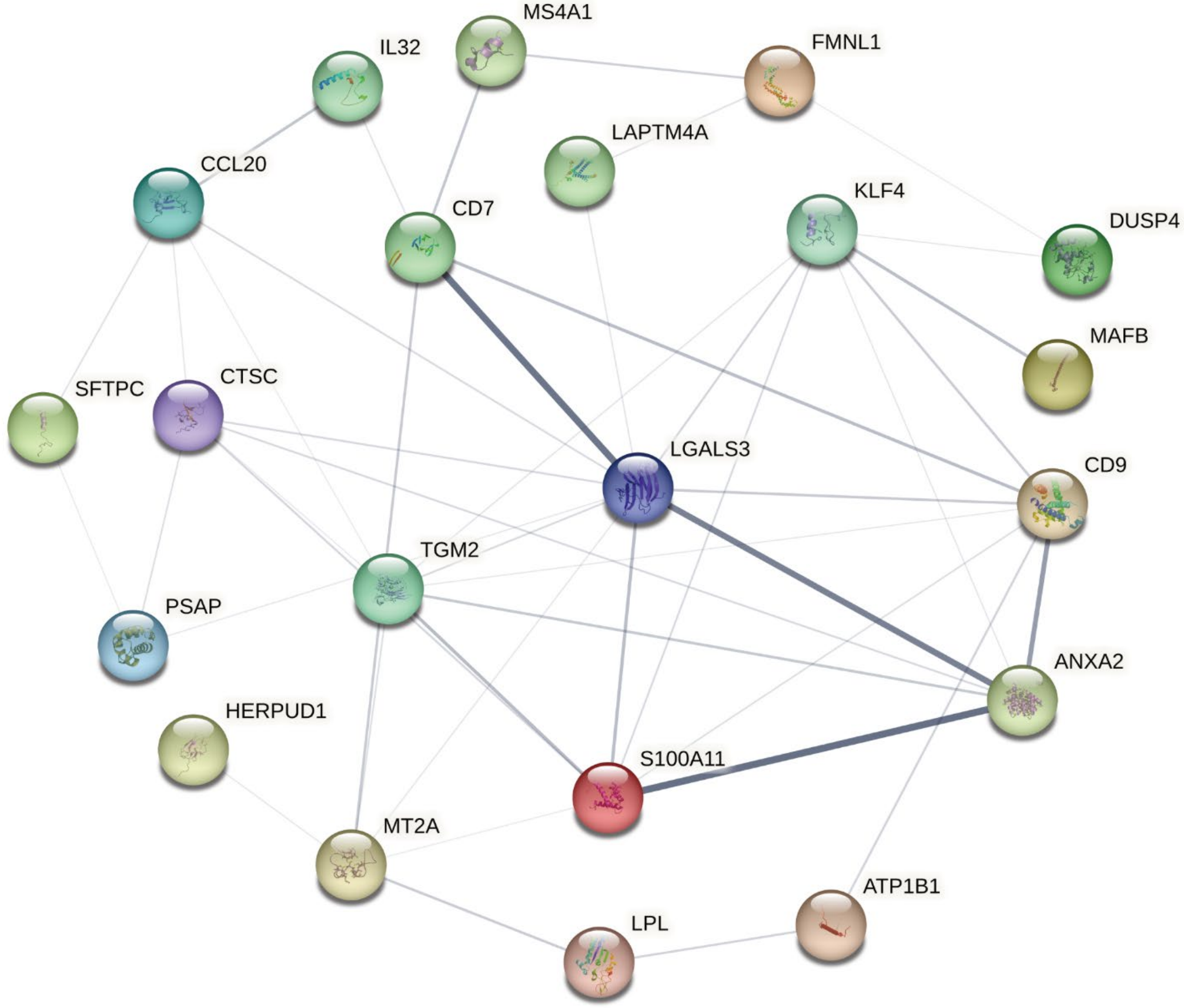

Supplementary Figure S3. A protein interaction network plot. (A) A protein interaction network was explored by a string database.

A

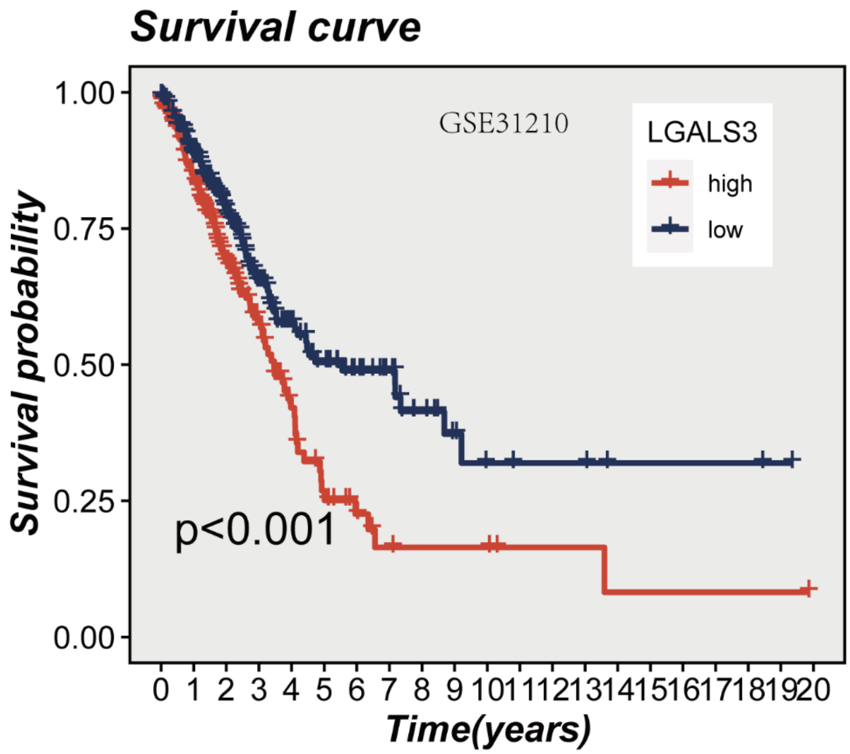

Supplementary Figure S4. Validation of the prognostic performance of LGALS3 in lung adenocarcinoma patients using the GSE31210 dataset.

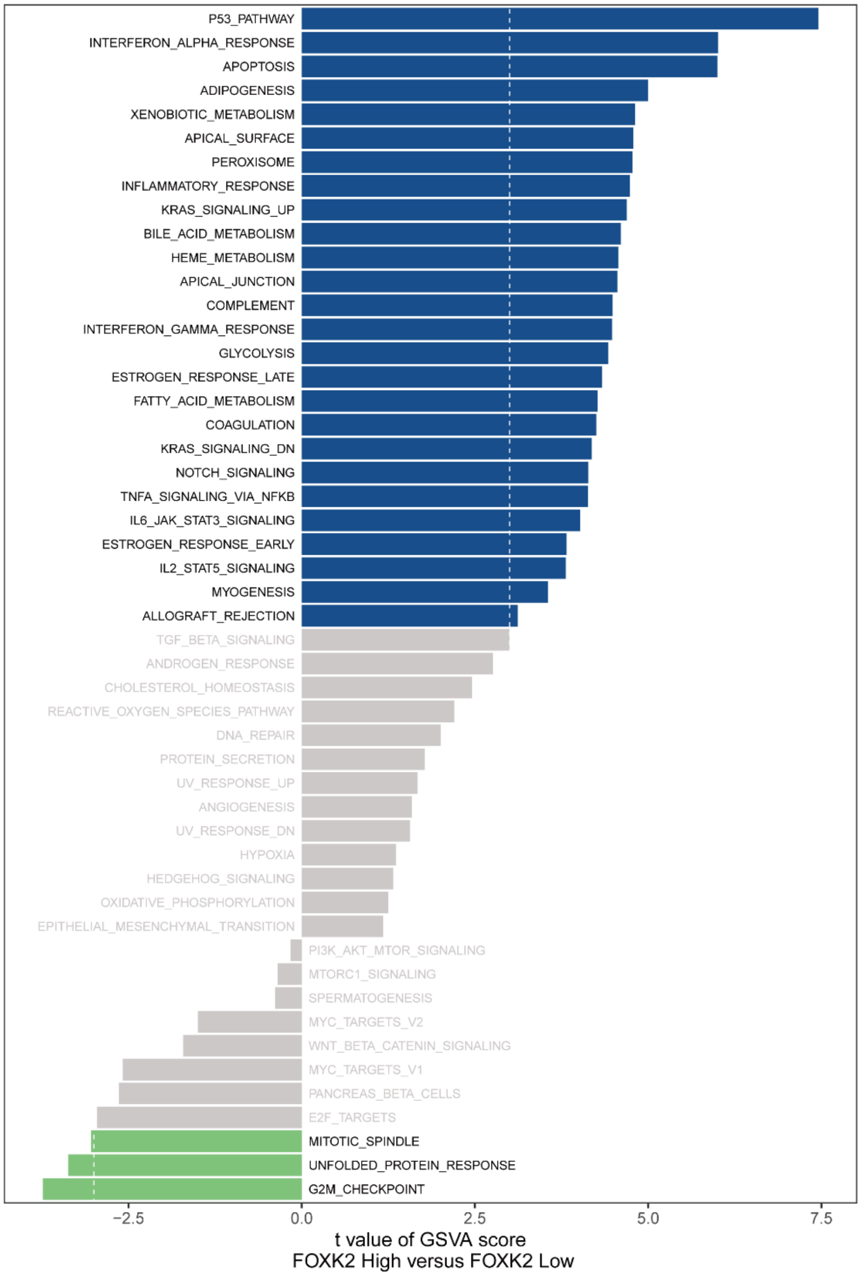

Supplementary Figure S5. GSVA enrichment analysis was performed using LGALS3 expression TCGA-LUAD patients divided into two groups with high and low expression.
