# Supplementary material for: Integrating multiple machine learning methods to construct glutamine metabolism-related signatures in lung adenocarcinoma
Source: Front Endocrinol (Lausanne). 2023 May 17;14:1196372. doi: 10.3389/fendo.2023.1196372 (PMC10229769; doi:10.3389/fendo.2023.1196372)
Supplement: Supplementary file 2 [file Table_1.docx]

| **Oligonucleotides** | **Nucleotide sequence (5'-3')** |
| --- | --- |
| **siRNA** |  |
| Scramble control | GCUUCGCGCCGUAGUCUUA |
| Si-LGALS3-1 | GCTCACTTGTTGCAGTACAAT |
| Si-LGALS3-2 | CCCACGCTTCAATGAGAACAA |
|  |  |
| **Primer** |  |
| GAPDH | GGCCTCCAAGGAGTAAGACC (forward) |
|  | AGGGGAGATTCAGTGTGGTG (reverse) |
| LGALS3 | GTGAAGCCCAATGCAAACAGA (forward) |
|  | AGCGTGGGTTAAAGTGGAAGG (reverse) |
|  |  |

**Table S1. Oligonucleotides used in research**
